# Factors Influencing Data Quality in Electronic Health Record Systems in 50 Health Facilities in Rwanda and the Role of Clinical Alerts: Cross-Sectional Observational Study

**DOI:** 10.2196/49127

**Published:** 2024-07-03

**Authors:** Hamish S F Fraser, Michael Mugisha, Ian Bacher, Joseph Lune Ngenzi, Christopher Seebregts, Aline Umubyeyi, Jeanine Condo

**Affiliations:** 1 Brown Center for Biomedical Informatics Brown University Providence, RI United States; 2 School of Public Health University of Rwanda Kigali Rwanda; 3 Jembi Health Systems Cape Town South Africa; 4 University of Cape Town School of Public Health Cape Town South Africa; 5 Center for Impact, Innovation and Capacity Building for Health and Nutrition (CIIC-HIN) Kigali Rwanda; 6 Tulane University New Orleans, LA United States

**Keywords:** data quality, electronic health record, EHR, electronic medical record, EMR, HIV, Rwanda

## Abstract

**Background:**

Electronic health records (EHRs) play an increasingly important role in delivering HIV care in low- and middle-income countries. The data collected are used for direct clinical care, quality improvement, program monitoring, public health interventions, and research. Despite widespread EHR use for HIV care in African countries, challenges remain, especially in collecting high-quality data.

**Objective:**

We aimed to assess data completeness, accuracy, and timeliness compared to paper-based records, and factors influencing data quality in a large-scale EHR deployment in Rwanda.

**Methods:**

We randomly selected 50 health facilities (HFs) using OpenMRS, an EHR system that supports HIV care in Rwanda, and performed a data quality evaluation. All HFs were part of a larger randomized controlled trial, with 25 HFs receiving an enhanced EHR with clinical decision support systems. Trained data collectors visited the 50 HFs to collect 28 variables from the paper charts and the EHR system using the Open Data Kit app. We measured data completeness, timeliness, and the degree of matching of the data in paper and EHR records, and calculated concordance scores. Factors potentially affecting data quality were drawn from a previous survey of users in the 50 HFs.

**Results:**

We randomly selected 3467 patient records, reviewing both paper and EHR copies (194,152 total data items). Data completeness was >85% threshold for all data elements except viral load (VL) results, second-line, and third-line drug regimens. Matching scores for data values were close to or >85% threshold, except for dates, particularly for drug pickups and VL. The mean data concordance was 10.2 (SD 1.28) for 15 (68%) variables. HF and user factors (eg, years of EHR use, technology experience, EHR availability and uptime, and intervention status) were tested for correlation with data quality measures. EHR system availability and uptime was positively correlated with concordance, whereas users’ experience with technology was negatively correlated with concordance. The alerts for missing VL results implemented at 11 intervention HFs showed clear evidence of improving timeliness and completeness of initially low matching of VL results in the EHRs and paper records (11.9%-26.7%; *P*<.001). Similar effects were seen on the completeness of the recording of medication pickups (18.7%-32.6%; *P*<.001).

**Conclusions:**

The EHR records in the 50 HFs generally had high levels of completeness except for VL results. Matching results were close to or >85% threshold for nondate variables. Higher EHR stability and uptime, and alerts for entering VL both strongly improved data quality. Most data were considered fit for purpose, but more regular data quality assessments, training, and technical improvements in EHR forms, data reports, and alerts are recommended. The application of quality improvement techniques described in this study should benefit a wide range of HFs and data uses for clinical care, public health, and disease surveillance.

## Introduction

### Background

By 2021, over 67% of people living with HIV were from sub-Saharan Africa [1]. Rwanda, along with other East African countries, has made great progress in the care and treatment of patients living with HIV/AIDS, including improvements in the uptake of prevention of mother-to-child transmission services and reduction in the rate of loss to follow-up for patients receiving antiretroviral therapy (ART). In 2019, a total of 84% of HIV-positive adults in Rwanda knew their status, 98% of those who knew their status were receiving ART, and 90% of those receiving ART had suppressed viral loads (VLs) [2]. HIV care has been available in Kigali (the capital) approximately from 2002. In 2005, partly through a collaboration between the Rwandan Ministry of Health (MOH) and the health care nongovernmental organization Partners in Health and Inshuti Mu Buzima (PIH-IMB), among other global partners, including the Centers for Disease Control and Prevention (CDC) and Global Fund, work began to scale up treatment to the whole population in need [3,4]. This scale-up treatment was designed around a community-based model, including routine care provision by nurses. Furthermore, it included the training and deployment of community health care workers for home follow-up and support and directly observed therapy for some patients. Ensuring long-term high-quality care for chronic diseases like HIV/AIDS also requires effective documentation of enrollment and follow-up care, with reliable access to clinical data at the point of care and quality improvement processes and reporting [5,6]. In 2005, PIH-IMB deployed a web-based medical record system, the HIV–electronic medical record (HIV-EMR), that had been developed for HIV care in Haiti [5]. These initial strategies were coupled with task shifting and scaling of diagnostics and treatment across Rwanda, reducing barriers related to geographical and financial access to the management of HIV/AIDS.

### Digital Health Electronic Health Records

In the last 5 years, Rwanda has initiated digital health as one of the 7 key pillars of the Smart Rwanda Master Plan, which involves the digitalization of different processes of health care delivery and management. Recently, significant efforts were made to deploy information systems and numerous digital tools to support various aspects of service delivery in the health sector [2] with the aim of reaching the *one citizen, one record* principle. The focus of building the national digital health enterprise architecture was to support seamless information exchange and the need to develop Rwanda Digital Health Interoperability Standards and Health Information Exchange, now known as OpenHIE.

Electronic health records (EHRs) are widely used for collecting, storing, viewing, analyzing, and sharing of clinical data. They address critical limitations in paper records that have been used for generations to support health care and are still relied upon in many lower-income countries. The potential benefits of EHRs over paper records include the following capabilities: unrestricted access to patients’ records for routine or emergency care, often in multiple locations; secure storage of data with backups; search and retrieval of patient records and key clinical data; clinical decision support to improve quality of care; generating reports; sharing data between facilities; disease surveillance; and clinical research. The success of these activities depends on the design and cost of the software; the availability of suitable hardware and infrastructure, particularly in low-income settings; and the ability and motivation of the staff to use the system [7-9]. Effective EHRs need to address important priorities of stakeholders (value of the system) [10,11] and be usable by staff as part of their routine work, which requires attention to the user interface, clinical workflow, and training [11-13] (ease of use). Regardless of the specific system in use, one metric is critical for creating value from the system in the form of data for decision-making processes: data quality [8,14]. If an EHR implementation cannot achieve and sustain good data quality, it is unlikely to create real value for stakeholders, whether they are clinical staff caring for patients, members of the health facility (HF) management, members of ministries of health conducting disease surveillance, or researchers studying questions such as disease incidence or management.

What constitutes good data quality depends on the use of the data: the principle of “data which is fit for purpose.” Clinical care requires data that are as complete and accurate as possible, but specific variables, such as medications, past medical history, or laboratory results, are often most important [15]. Missing data erode the value of the EHR and trust in the system. This typically leads to staff questioning the value of using the systems and keeping data up to date, which can lead to a downward cycle of deterioration and sometimes abandoning the EHR [9]. Conversely, a well-maintained system that is valuable for clinical tasks and reporting can incentivize better data entry. Many strategies have been used to improve data entry, including mapping the system’s workflow to the typical task order of staff, providing warnings of inappropriate values for a form field, and reporting values inconsistent with previous entries such as body weight or age. Training staff in system use and the importance of complete and up-to-date data are also priorities [16]. One essential strategy is monitoring data entry and completeness and periodic audits of data quality. As noted previously, data quality and completeness are typically linked. A fall off in data entry, for example, may be due to a technical issue or a lack of staff but can lead to gaps in the records and potential falls in data quality. Conversely, catching a lack of data entry quickly can identify correctable technical issues and allow early intervention to maintain momentum and confidence [17].

Many of these issues are more challenging in low- and middle-income countries (LMICs). In addition, the loss of reliable EHR data may have a greater impact on patient care and health system performance in LMICs, given the frequent lack of alternative records or strong health systems. Previous studies have investigated the benefits of different data entry and quality strategies, often in a small number of HFs [6,18]. However, many of these questions are most relevant in EHR systems deployed at scale and in longer-term use rather than pilot projects and recent deployments. There are important unanswered questions on what strategies are most effective at improving data quality, and what factors related to HFs and staff can facilitate or impede data quality [14]. Hedt et al [19] studied the quality of data in paper records of patients with HIV in Malawi using a technique termed lot quality assurance sampling (LQAS). Their study revealed high levels of completeness and accuracy of these records, all of which were reviewed by a supervising team on a quarterly basis. Furthermore, they studied how accurately the overall data quality could be predicted from a random sample of 76 records in each of the 19 HFs. This sample accurately predicted the overall data quality when a threshold of 85% on quality metrics was used to indicate a low quality.

### EHR Data Use for Clinical and Public Health

Routine clinical data, such as those collected from EHRs, are an important component of public health systems in high-income countries [20]. Moreover, the data in EHRs can help to understand the patterns of HIV distribution, the progress in achieving national and global targets, oversight of the risks and patterns in specific population groups, and the evolution of the infection in different circumstances [21,22]. EHR data shared across HFs may help to realize continuity of care as people move from one region to another. LMICs are increasingly adopting EHRs, with HIV care being in the vanguard in Eastern and Southern Africa, providing a test case for broader EHR adoption and use. The quality and timeliness of these data are a key factor in public health decision-making and management of programs to improve population health outcomes. EHR data are used for public health surveillance, patient to provider communication, quality of care monitoring, linkage of primary care and public health system such as notifiable disease reporting [23], and strategic policy direction [24]. Health Information Exchanges linking EHR and laboratory data, for example, have a growing role in population health and disease surveillance [25]. To maximize the benefits of EHRs for clinical and public health functions, they increasingly allow data collection on behavioral, social, and environmental factors that may affect patient and population health [26]. EHR systems in LMICs, such as OpenMRS, are able to interoperate with pharmacy information systems, laboratory information systems, and mobile health (mHealth) apps, including through the Health Level-7 Fast Healthcare Interoperability Resources (HL7 FHIR; Health Level Seven International) standard, creating more comprehensive views of patient care, population, and public health across a range of communities [27].

### The OpenMRS EHR

Recognizing the need for a general-purpose EHR system, PIH in collaboration with the AMPATH project (Academic Model Providing Access to Healthcare) in Eldoret, Kenya; the Regenstrief Institute in Indiana, United States; and the South African Medical Research Council deployed a new open-source general-purpose EHR for HIV care in 2006 [28]. This system, the OpenMRS EHR, was subsequently scaled to 43 PIH- IMB-supported HFs in Rwanda. From 2011 onward, it was rolled out to >300 MOH-run HFs. OpenMRS presently supports comprehensive services in all departments of district hospitals. Furthermore, it supports care for maternal-child health, noncommunicable diseases, and oncology in some health centers and hospitals in Rwanda and other countries [7]. OpenMRS is used in >44 LMICs [7], including support for HIV care in >5000 HFs, primarily in Kenya, Uganda, Mozambique, Nigeria, and Rwanda, and will soon be deployed widely in Ethiopia and Haiti [29]. OpenMRS continues to be upgraded with recent improvements in the user interface, data analysis and export, standardized clinical decision support systems, and improved interoperability using HL7 FHIR [7,27]. Previous studies on data quality in OpenMRS have been conducted [6], but only 1 published study by Muthee et al [14] evaluated data quality at scale in >50 HFs.

### Rwanda OpenMRS Evaluation

This study was part of a larger process evaluation and randomized controlled trial (RCT) of OpenMRS in Rwanda. The US CDC funded a process evaluation of the OpenMRS EHR system in use at scale in Rwanda. This included (1) a user survey, (2) key informant interviews, (3) monitoring of system use and technical stability, (4) a study of the costs of development and deployment of the enhanced EHR software, and (5) the data quality study described in this paper.

Furthermore, a cluster RCT on the impact of improving workflow and adding decision support tools on the quality of HIV care is currently being completed. Three types of alerts were implemented in the trial:

1. A warning if a patient newly diagnosed with HIV was not started on ART within 2 weeks (in the same clinic)
2. A warning for a missing VL result for patients after 6 months of HIV care and annually thereafter (with a 2-month window for test results to be returned and entered into the EHR)
3. A warning for abnormal VL results (>1000) suggesting virologic failure

A total of 112 HFs were included in the RCT, half of which (56/112, 50%) were randomized to receive alert 1; 28 (25%) of these HFs also received alert 2, and 14 (12.5%) of these also received alert 3. The alerts were triggered when clinicians opened the patient summary and were also presented as a report of all patients currently matching the rule. The end users were trained to interpret the alerts in the patient summaries and reports. The larger study design has been described previously [4].

We aimed to measure data completeness, matching, concordance, and timeliness and to evaluate factors influencing data quality and strategies to improve it. The data studied were sourced from OpenMRS, a widely deployed EHR system supporting HIV care in Rwanda for >15 years [7,30]. It builds on a clinical trial of the effects of clinical decision support tools on HIV care. Furthermore, it includes data on the characteristics of the HFs and the staff who work there, building on the results of an EHR user survey of the same group of facilities [4].

## Methods

### Overview

In this paper, we describe a cross-sectional study based on the LQAS approach used by Hedt et al [19]. A total of 50 (44.6%) HFs were selected from the 112 HFs included in the clinical trial, based on the power calculations for that study, selecting a random sample from both intervention and control HFs and a representative mix of larger and smaller HFs but not hospitals. Trained research assistants from the Rwanda School of Public Health visited the 50 HFs and collected data using Open Data Kit (ODK), an open-source mobile data collection software [31]. At each HF, depending on the patient volume, 48 to 76 charts were randomly selected from the set of patients receiving HIV care based on the LQAS protocol used by Hedt et al [19]. Data were extracted from both the paper and EHR records for each patient including 28 key data items on patient enrollment, ART, VL, medication pickup, and retention in care. Data were entered into the ODK app from both sources, and the dates for each variable were also recorded.

The deidentified data were uploaded to a study database housed at the University of Rwanda School of Public Health. Data analyses were partly based on the work of Hedt-Gautier et al in Malawi [19] and Rwanda on public health reporting systems and Muthee et al [14] on data quality analysis in the KenyaEMR OpenMRS-based system.

Analyses included the following metrics:

- Comparison of the paper records and EHR records for completeness of the data items
- Comparison of the paper records and EHR records for percentage of matching of the data items
- Concordance scores comparing the combined matches of 15 key variables
- Comparison of HFs that were recorded as having high EHR uptime and availability (in the previous user survey by Fraser et al [4]) to those with poor EHR uptime
- Comparison of HFs based on the duration for which staff have worked with the EHR and their level of technological experience
- Comparison of completeness of key variables in the EHR records of intervention HFs with those of control HFs,
  for records completed after the intervention was implemented

### Data Processing and Analysis

First, the data were processed to identify any missing values. Values were regarded as missing if the field was blank, was coded with an answer indicating that the information was not available, and, for date variables, if the date reported was impossible (ie, either too far in the future or dates related to HIV treatment that predated any HIV treatment occurring in Rwanda). The latter category helped address the fact that “missing” dates were often recorded as January 1, 1980. Then, for all 28 variables in the data set, we counted the number of records that did not have missing data in either the paper file or the EHR and recorded that value as the number filled or complete. This value was divided by the total number of records to obtain the percentage that were complete. The data were scored as “high quality” if ≥85% of the records were complete based on the LQAS protocol [19]*.*

Then, from the subset of records that were complete, we analyzed how many of them matched in both the paper file and the EHR. Before determining the number of matches, we performed basic data standardization. Specifically, drug regimens were sometimes reported as multiple combinations, for example, the medications could have been coded as Tenofovir (300) + Lamivudine (300) and Efevirenz (600) instead of Tenofovir (300) + Lamivudine (300) + Efevirenz (600); for these cases, we standardized the drug list to 1 standard code. For 22 data points (minus VL values), data were regarded as a match if the standardized value recorded in the paper file exactly matched the standardized value recorded in the EHR. Data quality for matches was regarded as “high quality” if 85% of the records that were complete matched in both the paper file and the EHR.

For each patient, *we also collected the last 3 VL results and dates, if available*. There were few “matching” records for VL results. Further analysis showed that this usually occurred when the paper file had 1 newer VL result than the EHR; therefore, the most recent VL result in the EHR exactly matched the second most recent VL result in the paper file. To account for this, we aligned the records based on date; for example, if the EHR had a record for May 1, 2019, this would be treated as being the same entry as a record from the paper file that was also dated May 1, 2019. VL results recorded with a date in the paper file that did not match the date of a recorded VL result in the EHR were counted as missing in the EHR and vice versa. Because we only had the last 3 records from each file, in any case where the paper record had newer data than the EHR, there were always at least 2 missing records. To account for this, we aligned records in the EHR and paper file based on date and produced 2 derived variables: “All Viral Loads,” which summarizes all VL records but counts matches based on date rather than position, and “At Least 1 Viral Load,” which examines whether the most recent value in the EHR matches one of the 3 values in the paper file. Data completeness for filled VLs was regarded as “high quality” if ≥85% of the records existed in both files.

After the date alignment was completed, we standardized values such that VL numbers with a small difference within 1 copy/mL were counted as a match. This helped address cases where VL results were reported with additional precision (20.6 vs 20 or 19.2 vs 20), noting that the EHR system only recorded absolute numbers (ie, 20 instead of 20.6). *For all filled VLs (VLs where the date matched in both the paper file and the EHR), we counted them as a match if the standardized VL numbers in both records matched*. VL matches were regarded as “high quality” if 85% of the records matched in both the paper file and the EHR.

Subsequently, we performed the same analysis for the data *broken down by individual health care facilities,* allowing for the analysis of potential factors affecting data quality. First, we examined the correlations between the HF characteristics and the resulting data quality. Facility characteristics were obtained from the user survey collected at the same time as this data quality study [4]. We hypothesized that the relevant factors would be (1) whether the HF was an intervention or control HF, (2) the average time (years) that the clinical users and data managers had been working with the EHR, (3) the relative technology experience of the users, and (4) how available and stable the EHR was. We hypothesized that being an intervention HF, the length of time of working with the EHR, EHR uptime, and technical experience would be positively correlated with better data quality.

To measure overall data quality, we developed a *concordance score* based on the work of Muthee et al [14] who compared data quality in paper and EHR records in Kenya. We narrowed the number of variables to 15 by considering only the most recent VL and drug pickup results and by dropping the variables for the health center sector, district, and facility, as these were not patient-specific data. The dropped variables were highly correlated or had little data entered in the paper file or EMR. The concordance score was calculated per record as the number of variables that exactly matched in both the paper file and the EHR after the values had been normalized, following the approach of Muthee et al [14]. *This differed from our above mentioned analysis in that if the data were missing in both the paper file and the EHR, the records were counted as concordant.* Therefore, the concordance score here is a number from 0 to 15, which is the count of the number of concordant variables in a single record.

The randomization list of the control and intervention arm HFs was used to label the HFs in this study as intervention or control. For the number of years of experience with the EHR, we used the arithmetic mean of the number of years the clinical users and data managers at each HF had been using the EHR (if there was >1 individual). Technology experience was determined based on answers to the survey regarding “outside of work” use of technology, which included how frequently they (1) sent SMS text messages, (2) used the internet on a mobile phone, (3) used a computer, or (4) accessed the internet. For each question, they received 1 point for each time they said they used the technology either “most of the time” or “always,” resulting in a score from 0 to 4.

### Statistical Analysis

The study metrics are summarized in Textbox 1. We calculated the correlation between the concordance score and each HF characteristic using the Pearson correlation coefficient with Bonferroni correction applied to adjust for multiple comparisons. Subsequently, we sought evidence for the *hypothesis that being an intervention site in the RCT would have a positive effect on the recording of VLs or drug dispensing episodes in the EHR*. VL results and drug regimens were standardized as described in the *Data Processing and Analysis section* (due to different ways of recording the same data) and then counted as a match when the data were not missing and matched exactly. We could only compare data from after the intervention had been rolled out in July 2018. We then evaluated whether there was any association between the variables using a simple chi-square test to determine whether there was a correlation and using a Pearson contingency coefficient to estimate the effect size (for simple data such as a 2×2 contingency square, Pearson contingency is equivalent to both the phi coefficient and Cramer V). Data analyses were performed using R software (R Foundation for Statistical Computing).

**Metrics defined in the study.**

**Metric and definition**

- Data completeness
  Number of fields filled divided by all fields
- Data matching
  Number of fields in the electronic health records that match those in the paper records divided by the number of filled fields in the paper records
- Concordance scores
  Number of variables among the 15 selected variables in which the electronic health records and paper records match in each medical record (including empty fields in both record types; Data matches and concordance scores were based on data that had been normalized as described in the Methods section)
- Impact of hospital facility factors on concordance scores
  The comparison of concordance scores in hospital facilities with and without the hospital facility characteristic (eg, high electronic health record uptime)

### Ethical Considerations

The study was approved by 3 institutional review boards: Rwanda National Ethics Committee, Kigali (913/RNEC/2016), and the University of Leeds School of Medicine Research Ethics Committee, Leeds, United Kingdom (MREC16-176). This study was also reviewed in accordance with the US CDC human research protection procedures (approval no CGH HSR 2014-270a) and determined to be research. However, CDC investigators did not interact with human subjects or have access to identifiable data during this study.

## Results

### Overview

A total of 3467 records were reviewed in both the paper charts and the EHRs in the 50 HFs selected, with a mean of 69.3 (SD 8.8) records reviewed per HF. A total of 194,152 data items were collected using the ODK-based study tool. Three metrics of data quality were applied: (1) data completeness of EHR and paper records, (2) matching of the values of individual data types between EHR and paper records, and (3) concordance scores of matches for 15 regularly collected data items between EHR and paper records.

Table 1 presents all 28 variables collected from the paper and EHR records and the percentage completeness of each category. Few patients were on second-line drugs and possibly 1 was on third-line drugs (shown in the EHR only) therefore these 4 fields were not included in further analysis. Notably, paper VL records were 20% to 30% more complete than EHR records.

Table 2 presents the matching of variables between the paper records and the EHR records. Most nondate variables had fairly high levels of matching, although 5 were between 82% and 85%, just below the threshold set for high quality. Similar to the data completeness results, VL values had lower matching rates (59.5% for the most recent value and 65.8% for the oldest value). Few patients were prescribed second-line or third-line drug regimens (and their matching was low).

Additional analysis of date matches showed that for single-entry dates, such as “date of enrollment in HIV care,” matches were fairly high (60.2%-75%) compared to repeated dates, such as “date of last visit” or drug pickup date. For the “one-off” dates, most errors appeared to be mistakes in transcription (eg, small date differences and single character errors). Detailed analysis of the degree of error in these date fields showed that many incorrect one-off dates, such as “date of enrollment in HIV care,” were within 1 month of the correct value 34% to 38% of the time. Conversely, for regularly updated dates, only 10% to 13% of the errors were within 1 month of the correct value or 29% to 34% of the errors were within 3 months, suggesting that these items were not updated frequently in the EHRs.

**Table 1:** Completeness of all 28 variables^a^ in the paper records and electronic health records (EHRs) and the identification of variables included in the concordance score (N=3467).

Number	Variable name	Variable included in concordance score	Completeness in paper records, n (%)	Completeness in EHR records, n (%)
1	Date of birth	Yes	3465 (99.94)	3466 (99.97)
2	Gender	Yes	3441 (99.25)	3467 (100)
3	Health center district	No	3467 (100)	3467 (100)
4	Health center sector	No	3467 (100)	3467 (100)
5	Health center name	No	3467 (100)	3467 (100)
6	Date of last visit	Yes	3464 (99.91)	3465 (99.94)
7	Date of first HIV-positive test	Yes	2711 (78.19)	2273 (65.56)
8	Date of enrollment in HIV care	Yes	3456 (99.68)	3457 (99.71)
9	Date of first prescribed ARTs^b^	Yes	3463 (99.88)	3383 (97.58)
10	Initial WHO^c^ stage	Yes	3433 (99.02)	3267 (94.23)
11	Regimen 1 start date	Yes	3404 (98.18)	3385 (97.63)
12	Regimen 1 drugs	Yes	3411 (98.38)	3311 (95.50)
13	Regimen 2 start date^d^	Yes	135 (3.89)	86 (2.48)
14	Regimen 2 drugs^d^	Yes	136 (3.92)	86 (2.48)
15	Regimen 3 start date^d^	No	0 (0)	1 (0.03)
16	Regimen 3 drugs^d^	No	0 (0)	1 (0.03)
17	Drug pickup 1 date	Yes	3466 (99.97)	3465 (99.94)
18	Drug pickup 1 drugs	Yes	3466 (99.97)	3312 (95.53)
19	Drug pickup 2 date	No	3433 (99.02)	3385 (97.63)
20	Drug pickup 2 drugs	No	3434 (99.05)	3233 (93.25)
21	Drug pickup 3 date	No	3395 (97.92)	3267 (94.23)
22	Drug pickup 3 drugs	No	3397 (97.98)	3123 (90.08)
23	Viral load 1 date	Yes	3280 (94.61)	2645 (76.29)
24	Viral load 1 value	Yes	3284 (94.72)	2649 (76.4)
25	Viral load 2 date	No	2951 (85.12)	1773 (51.14)
26	Viral load 2 value	No	2955 (85.23)	1775 (51.19)
27	Viral load 3 date	No	2382 (68.7)	1116 (32.19)
28	Viral load 3 value	No	2386 (68.82)	1118 (32.25)

^a^All variables met the 85% threshold except date of first HIV-positive test, viral load data, and second-line and third-line drug regimen data.

^b^ART: antiretroviral therapy.

^c^WHO: World Health Organization.

^d^Very few participants had the records for these variables.

**Table 2:** Matching of the variables between the paper records and the electronic health records (EHRs; N=3467). Several variables were just below the 85% threshold.

Variable name	Complete in paper record and EHR, n (%)	Matches, n (%)
Date of birth	3464 (99.91)	3242 (93.59)
Gender	3441 (99.25)	3326 (96.66)
Health center district	3467 (100)	3452 (99.57)
Health center sector	3467 (100)	3330 (96.05)
Health center name	3467 (100)	3467 (100)
Date of last visit	3462 (99.86)	979 (28.28)
Date of first HIV-positive test	1830 (52.78)	1102 (60.22)
Date of enrollment in HIV care	3446 (99.39)	2583 (74.96)
Date first prescribed ART^a^	3379 (97.46)	2443 (72.3)
Initial WHO^b^ stage	3236 (93.34)	2740 (84.67)
Regimen 1 start date	3322 (95.82)	2382 (71.7)
Regimen 1 drugs	3256 (93.91)	2673 (82.09)
Regimen 2 start date	78 (2.25)	42 (53.85)
Regimen 2 drugs^c^	78 (2.25)	37 (47.44)
Regimen 3 start date	0 (0)	0 (0)
Regimen 3 drugs	0 (0)	0 (0)
Drug pickup 1 date	3464 (99.91)	986 (28.46)
Drug pickup 1 drugs	3311 (95.5)	2772 (83.72)
Drug pickup 2 date	3363 (97)	698 (20.76)
Drug pickup 2 drugs	3212 (92.64)	2697 (83.97)
Drug pickup 3 date	3220 (92.88)	541 (16.8)
Drug pickup 3 drugs	3078 (88.78)	2583 (83.92)
Viral load 1 date	2614 (75.4)	847 (32.4)
Viral load 1 value	2622 (75.63)	1559 (59.46)
Viral load 2 date	1719 (49.58)	547 (31.82)
Viral load 2 value	1725 (49.75)	1095 (63.48)
Viral load 3 date	1033 (29.8)	322 (31.17)
Viral load 3 value	1038 (29.94)	683 (65.8)

^a^ART: antiretroviral therapy.

^b^WHO: World Health Organization.

^c^The only matching rate below 59% that was not a date was for regimen 2 drugs that had very little data (n=78).

### Concordance Scores

The mean overall concordance score was 10.2 (SD 1.28) for 15 (68%) variables in all 50 HFs. Only 2 HFs were at or above the threshold of 85% concordance. However, 8 of the 15 variables in the concordance score (not including the date of birth) were dates (refer to Table 1 for the included variables). The concordance score would be higher if dates that match within 1 month were included.

### Evaluation of Factors Associated With Concordance Scores in Each HF

**Figure 1:**
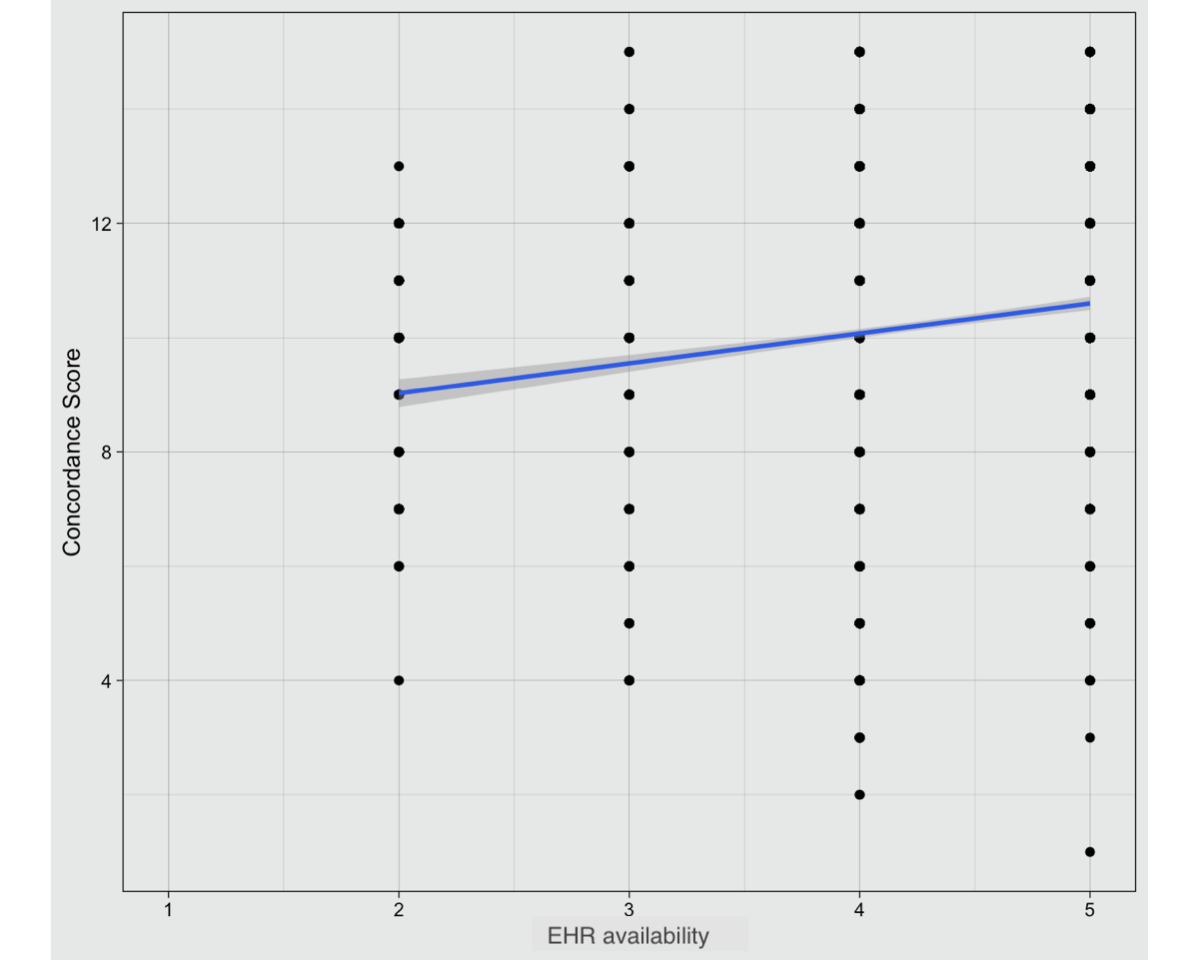
Correlation between concordance scores and the reported electronic health record (EHR) availability or uptime (ranging from 1 to 5 Likert scale responses of EHR users).

Correlations between the concordance scores and most HF characteristics were not significant. For the survey response that “the EHR was available always or nearly always,” there was a positive correlation with the *concordance score*, with *r*=0.16 (95% CI 0.13-0.19; *P*<.001; Figure 1). There was also a significant (negative) relationship between user responses suggesting that they had higher *technology experience,* and *concordance* scores with *r*=−0.11 (95% CI –0.15 to 0.08; *P*<.001). There was no significant correlation between the *concordance scores* and users’ reported years of EHR use (Table 3).

**Table 3:** Association of electronic health record (EHR) performance and use characteristics with concordance scores.

Parameter 1	Parameter 2	*r* (95% CI)	*t* test (*df*)	*P* value
Concordance score	EHR availability	0.16 (0.13 to 0.19)	9.37 (3326)	<.001
Concordance score	Technology experience	−0.11 (−0.15 to −0.08)	−6.64 (3326)	<.001
Concordance score	Years of EHR use	−0.04 (−0.07 to 0.00)	−2.10 (3326)	.21

### Effects of Alerts in the Intervention HFs on Matching of Key Variables in EHRs and Paper Records

**Figure 2:**
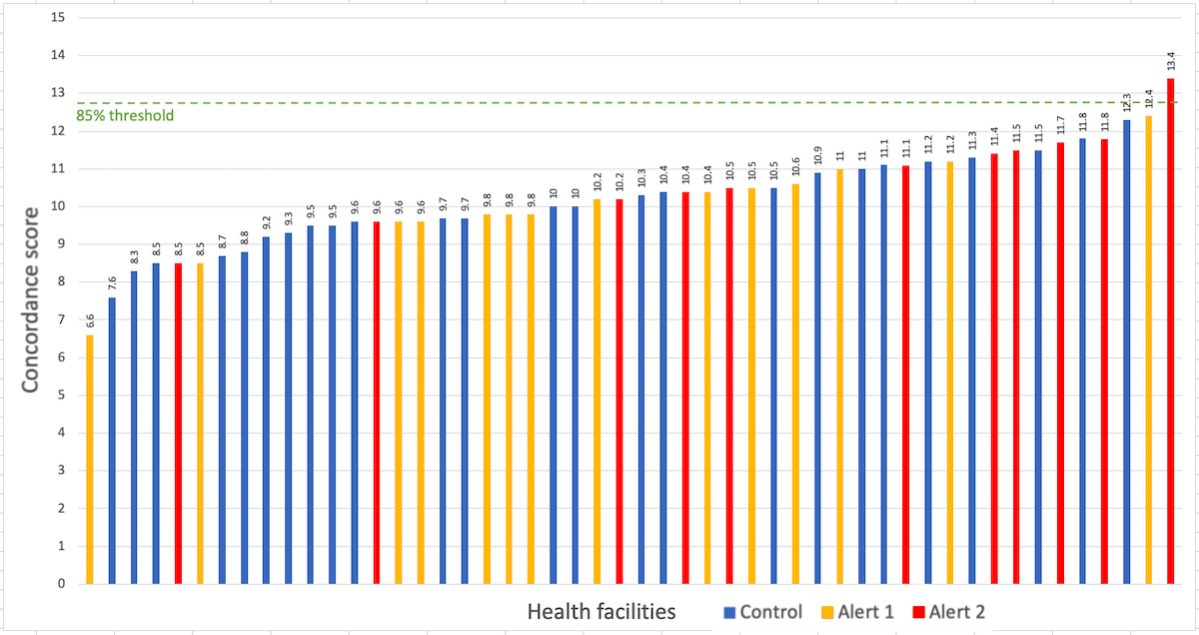
Mean concordance scores for all health facilities color coded by intervention status (maximum score=15).

**Figure 3:**
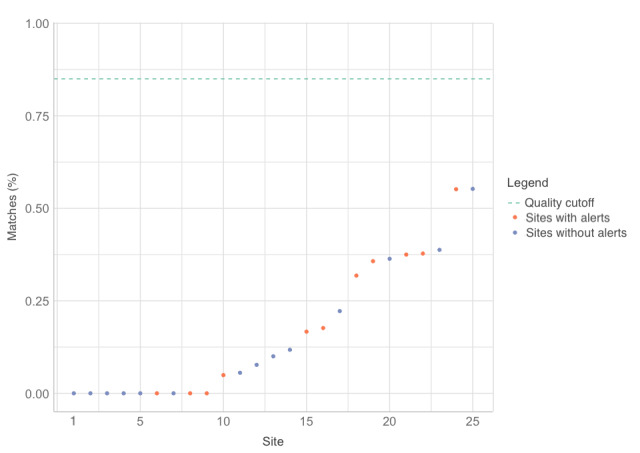
Graph showing percentage data matches for viral load results between paper records and electronic health records for each of the 25 health care centers in the intervention arm, ranked by percentage of matches.

As the intervention was implemented in July 2018 and the data for the data quality study were collected in November and December 2018, there was a 4-month to 5-month window when the alerts were active. All intervention HFs had alerts for *starting a new treatment*, but few *new patients* were enrolled in the 5-month time frame. Therefore, there was insufficient data to study the effects of alert 1. However, there was sufficient data to compare the data quality metrics of the 11 intervention HFs that had *alerts for missing VL results* (alert 2) with the 39 HFs without these alerts, Figure 2 shows the concordance scores for each site color coded by intervention status. Figure 3 presents the effect of the VL alerts on overall data matches between the EHRs and the paper records, ranked by percentage matching of variables between the 2 sources. The HFs with the VL alerts (orange color) had a higher rank overall than the control HFs.

In HFs with alerts, 85 (26.7%) of 318 VL records in the EHR matched those in the paper records, but in HFs without alerts, only 120 (11.9%) of 1008 VL records matched (*P*<.001, chi-square test)

**Figure 4:**
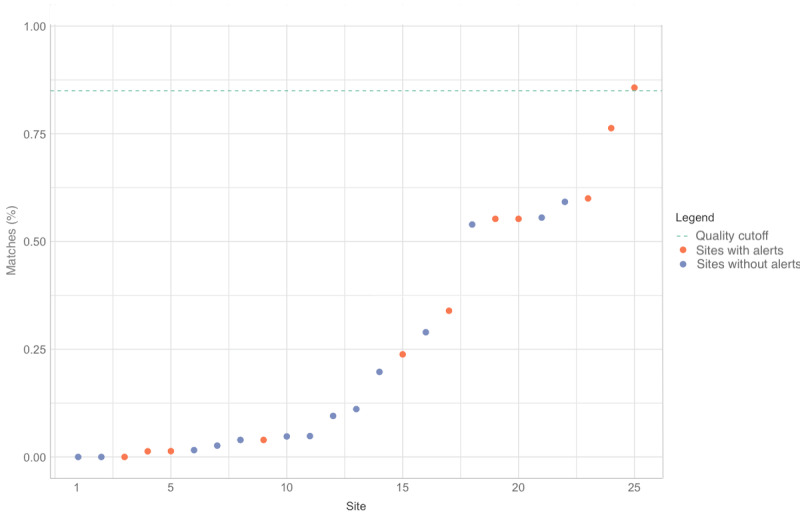
Graph showing percentage data matches for drug pickups between paper records and electronic health records among the same 25 intervention health facilities (HFs) as in Figure 3. The overall scores were higher than in Figure 3 but only 1 (intervention) HF score was more than the data quality threshold.

Furthermore, we *compared the number of drug pickups in the EHRs that exactly matched with those in the paper file records*, comparing the intervention HFs with VL alerts with HFs without such alerts. The results are shown in Figure 4. In HFs with alerts, 280 (36.2%) of 774 of the records of drug pickups matched, but in HFs without alerts, only 183 (18.7%) of 981 matched (*P*<.001). A second analysis using the chi-square test and Pearson contingency coefficient for the 25 HFs revealed χ^2^_1_=67.5 (*P*<.001), indicating a strong effect (*ϕ*=0.20, 95% CI 0.15-0.24). Despite the significantly higher data quality at the intervention HFs, not all HFs showed this effect.

## Discussion

### Principal Findings

The results showed that the completeness of the paper records and EHR records was generally >85% threshold for good quality, with the exception of VL data collected in the 2 years before the study data collection (VL results 2 and 3). The completeness varied across the data items, with the best results (>99%) for EHR data seen in demographic data, date of enrollment in antiretroviral (ARV) care, date of first being prescribed ARV drugs, and date of last visit. The lowest completeness was seen in the second-line and third-line drug regimens, but very few patients were prescribed these regimens. There was clear evidence that the electronic records were not being updated in a complete and timely manner for some data items at most HFs (based on concordance scores), with only 2 HFs meeting the 85% threshold for good data quality. Furthermore, there was evidence that certain HFs were more effective in data entry and management. Using data from the previously published user experience survey by Fraser et al [4], we compared the measures of user experience with the EHRs in the same HFs included in this study. Facilities reported to have more consistent uptime and availability of EHRs showed significantly better data quality based on the concordance scores. However, HFs with higher “technology experience” scores had lower concordance scores, the reason for which is unclear.

A key scientific question for the overarching study was to determine whether the enhanced EHR in the intervention HFs improved both the usability and use of the EHR and the completeness and overall quality of key data items required to support effective HIV care. The second decision support alert (refer to the Methods section) was designed to warn if a VL result had not been uploaded to the system by 6 months after starting ARV treatment or by 12 months after the last VL result (allowing for up to 2 months transport and data entry delays). There was a 4-month to 5-month period with the intervention in place before we collected data for this data quality study, allowing the assessment of whether VL data were more complete in the EHRs from the 11 HFs that received this alert. Although data completeness and timeliness for VL data were overall <85% of the threshold, with only 11.9% completeness in the 39 control HFs lacking the VL alert, completeness was significantly higher (26.7%) in the 11 intervention HFs that had VL alerts. A similar effect was seen for drug pickup data, suggesting that the presence of VL alerts impacted the overall data entry processes or behavior and not just the specific data type in the alert. It should be noted that VL testing was infrequent before the start of the study, as the laboratory capacity was still being established, which may have affected data management and data entry in the EHRs, especially for older values.

The metrics for overall data quality and completeness may have been higher if there had been regular data quality improvement visits to Rwandan MOH HFs, an effect seen in the study by Muthee et al [14] in Kenya. Another strategy for improving data quality and user experience is the implementation of EHR systems, allowing direct point-of-care data entry rather than entering data on paper and in the EHR, as observed for VL results in Kenya [32]. The findings in this study also indicate that data quality and timeliness would have likely been improved more generally using automated tools to alert staff of missing data for key variables such as VL results, using form field validation to detect impossible values, and comparing date values with other data. The analysis of the degree of error in date fields showed that many incorrect one-off dates, such as “date of enrollment in HIV care,” were within 1 month of the correct values for approximately one-third of the times and therefore may have been adequate for the care of HIV patients seen every 2 to 3 months. Incorrect repeated dates such as “last clinic visit,” “drug pickup,” and “VL” were within 1 month of the correct date, ≤12% of the times, and therefore may be less fit for use in patient care. Overall, most data were considered fit for purpose, as confirmed by the user survey [4], which showed a reliance on the EHR data for clinical care and reporting. Regular monitoring of data quality metrics and intervention at poorly performing HFs would likely help address major deficiencies efficiently.

The implications of these results are much broader than just HIV care, which provided the environment for this study. Primary care EHR systems remain rare in LMICs, but recent progress with facility-based EHR systems, such as OpenMRS in Haiti, Sierra Leone, parts of India, and the Philippines, are laying the foundations for wider use [33]. Mobile-based and tablet-based systems that support care in communities and some HFs should provide a much wider range of data for clinical and public health use. The World Health Organization (WHO)–led Open Smart Register Project [34] has 150 million patients now, the majority being in Bangladesh. Other mHealth systems, such as CommCare [35] and ODK [31], are used in many LMICs [36]. All these systems have the ability to share data with EHRs, such as OpenMRS. They increasingly support interoperability standards such as HL7-FHIR [27] for interoperability with a range of other health information systems, including laboratory and pharmacy systems, radiology information systems, and the District Health Information System-2 [37]. FHIR-based Application Programming Interfaces and toolkits are also being built into the Apple OS and Android operating systems [36]. Projects using data science techniques and machine learning are increasing in LMICs and require high-quality data sets that are representative of all communities and groups to avoid biases or poor performance in vulnerable groups [38].

### Comparison to Previous Work

Muthee et al [14] studied data quality in 53 HFs running the KenyaEMR OpenMRS-based system. They analyzed data from a data quality assessment (DQA), which had been collected by MOH lead teams that visited the HFs. They found ≥1 missing value in 735 (31%) paper forms and 747 (32%) KenyaEMR records, a higher missing rate than that in this study. Concordance scores were calculated in the same manner as in this Rwanda study, with a mean concordance of 11.9 (SD4.0) for 20 variables at baseline. Furthermore, they were able to compare the DQA results with a second DQA assessment of 27 HFs. They showed that HFs with previous DQA had significantly better data quality on the second visit, with missing data falling to 13% and concordance scores increasing to a mean of 13.6 (SD 4.2, difference in concordance scores *P*=.02). This result suggests that the process of measuring data quality and providing feedback to staff can have long-term benefits. The mean concordance scores of 13.6 (SD 4.2) for 20 variables (68%) at the second DQA were equivalent to those in Rwanda, which was 10.2 (SD 1.28) for 15 variables (68%). Abiy et al [39] studied data quality in an HIV treatment program in Ethiopia. They compared the paper and EHR versions of 250 records. Data completeness was 78% (95% CI 70.8%-85.1%) for paper records and 76% (95% CI 67.8%-83.2%) for the EHR. They used the κ statistic to compare variables in both records, with results ranging from κ=0.93 for demographics, κ=0.86 for WHO stage, and κ=0.83 for “general appearance.” Furthermore, Ngugi et al [40] studied the clinical use and completeness of data entry in KenyaEMR at 219 HFs in Kenya. They showed a wide variation in data entry per month in different HFs and noted that this was likely affected by “patients’ volume, frequency of patients’ visits (encounters), EHRs mode of use, and active use of the system during care.” Haskew et al [41] studied the quality of data collection and the effects of clinical alerts on HIV patient care in Western Kenya before and after the cloud-based implementation of an OpenMRS EHR. They showed that clinical alerts reduced missing data and improved the quality of care. Missing data were significantly reduced for key variables (before and after alerts implemented) including “patient source” (375 to 69 patients), “first CD4 count” (826 to 354 patients), and “first WHO stage” (2258 to 479 patients; all *P*<.001). The number of patients eligible for ART (based on CD4 count and WHO stage) but not yet started receiving it, fell from 1346 (29.6%) to 270 (6.2%; *P*<.001) [41].

A study of the health management information system in 16 health centers, 28 health posts, and 1 hospital in Ethiopia showed that training staff to fill out the data forms was significantly associated with improved data quality (odds ratio [OR] 2.253, 95% CI 1.082-4.692), and measures showing effective supervision and leadership were also beneficial [42]. A study of HIV clinical data collection systems at 21 HFs (18 in Africa) showed a reduction in missing data associated with training in data management (OR 0.58, 95% CI 0.37-0.90) and weekly hours spent by a data clerk working on the data collection system (OR 0.95, 95% CI 0.90-0.99) [16]. Previous studies suggest that poor completion or accuracy of key data items required for clinical care is likely to limit the benefit of EHR systems in day-to-day patient management, such as the functioning of clinical decision support system in this study and that reported by Haskew et al [41]. Furthermore, it impacts quality of care metrics (eg, in cancer care in the United States [43]) and efficient reporting of data for clinical teams, MOH, and funders [16,42].

### Limitations

The study was limited to a subset of the MOH-run OpenMRS EHR systems for use in HIV care. The inclusion criteria for the 112 HFs in the larger study favored HFs with better information technology hardware and successful implementation of a server monitoring tool [4]. Although the intervention HFs appeared to show improved data quality and completeness for certain variables, this effect might change over time. Although the user survey showed that staff in the intervention and control HFs received similar amounts of training and were equally positive about their training, it is possible that differences in the type of training in the intervention HFs and emphasis on the importance of VL contributed to better data quality. More robust and longitudinal studies are needed to understand the effects of different technologies on the use of EHR, including variations within and across HFs and individuals.

### Conclusions

The study found that there was generally high data completeness in both EHR and paper records in Rwandan MOH-supported HFs. The percentage matching and concordance scores for the EHR and paper records were lower, but this mainly affected the recording of dates. The considerable potential benefits of EHR systems for patient care for HIV and other diseases will only be realized with sufficient support, training and monitoring of data quality, and adequate technical support and infrastructure to ensure reliable systems. The use of “point-of-care” EHR systems rather than transcription of data from paper records can also improve user experience and data completeness. The results of this study show that automated alerts regarding data quality, completeness, and timeliness can significantly improve these metrics in remote HFs in a low-income country. However, to be fully effective, these alerts should be combined with other strategies to ensure high system uptime and a range of data quality improvement strategies, including training, regular supervision, and feedback. These improvements will likely help drive the much wider use of EHR data in LMICs for clinical care, reporting, data science, and machine learning. Furthermore, the data collected will support population and public health uses, including syndromic surveillance, notifiable disease reporting, real-time monitoring of disease burden, and forecasting resource requirements to meet care needs.

## Abbreviations

ART: antiretroviral therapy
CDC: Centers for Disease Control and Prevention
DQA: data quality assessment
EHR: electronic health record
EMR: electronic medical record
HF: health facility
HL7 FHIR: Health Level-7 Fast Healthcare Interoperability Resources
IMB: Inshuti Mu Buzima
LMICs: low- and middle-income countries
LQAS: lot quality assurance sampling
MOH: Ministry of Health
ODK: Open Data Kit
OR: odds ratio
PIH: Partners in Health
RCT: randomized controlled trial
VL: viral load
WHO: World Health Organization

## Data Availability

The data sets generated and analyzed in this study are available from the corresponding author upon reasonable request.
